# The topographical distribution of spermatogonial subpopulations during the cycle of seminiferous epithelium in *Macaca Fascicularis*

**DOI:** 10.1093/biolre/ioag018

**Published:** 2026-01-21

**Authors:** Martina Palazzoli, Chiara Capponi, Sara Di Persio, Stefania Fera, Antonio Filippini, Stefan Schlatt, Nina Neuhaus, Elena Vicini

**Affiliations:** Department of Anatomy, Histology, Forensic Medicine and Orthopedic, Section of Histology, Sapienza University of Rome, 00161 Rome, Italy; Department of Anatomy, Histology, Forensic Medicine and Orthopedic, Section of Histology, Sapienza University of Rome, 00161 Rome, Italy; Department of Obstetrics, Gynecology, and Reproductive Sciences, University of California, San Diego, La Jolla, CA, USA; Department of Anatomy, Histology, Forensic Medicine and Orthopedic, Section of Histology, Sapienza University of Rome, 00161 Rome, Italy; Centre of Reproductive Medicine and Andrology, University Hospital Münster, 48149 Münster, Germany; Department of Anatomy, Histology, Forensic Medicine and Orthopedic, Section of Histology, Sapienza University of Rome, 00161 Rome, Italy; Department of Anatomy, Histology, Forensic Medicine and Orthopedic, Section of Histology, Sapienza University of Rome, 00161 Rome, Italy; Centre of Reproductive Medicine and Andrology, University Hospital Münster, 48149 Münster, Germany; Centre of Reproductive Medicine and Andrology, University Hospital Münster, 48149 Münster, Germany; Department of Anatomy, Histology, Forensic Medicine and Orthopedic, Section of Histology, Sapienza University of Rome, 00161 Rome, Italy

**Keywords:** spermatogonial stem cell, spermatogenesis, seminiferous epithelium, stem cell niche, male infertility

## Abstract

The maintenance of mammalian spermatogenesis depends on the intricate molecular and cellular interactions between spermatogonial stem cells and their cognate niche in the seminiferous epithelium of the testis. To sustain the continuous production of sperm, spermatogonia proliferate and differentiate under the control of various niche factors, promoting either self-renewal or commitment to spermatogonial differentiation. Single-cell RNA sequencing analyses have identified different subpopulations of spermatogonia in primates based on the expression of specific marker genes (PIWIL4, GFRA1, NANOS3, and KIT). However, the spatial distribution of the different spermatogonial subpopulations and their relationship with the niche has not been described yet. Here, we investigate the topological localization of spermatogonia in primates. To this end, immunohistochemical stainings for PIWIL4, GFRA1, NANOS3 and KIT were performed on Bouin fixed samples of Macaca fascicularis and quantitatively analyzed. Strauss linear selectivity index (Linear Index, Li) was employed to assess the regional distribution of spermatogonial subpopulations in the basal compartment of seminiferous tubules. Remarkably, PIWIL4+ spermatogonia showed a random distribution along the basal compartment across all the stages of the seminiferous epithelium cycle. In contrast, GFRA1+, NANOS3+, and KIT+ spermatogonia displayed stage-dependent localization patterns. The spatial organization of different spermatogonial subpopulations, appeared coordinated with the cycle of the seminiferous epithelium, suggesting a dynamic regulation of spermatogonial behavior throughout the process of sperm production. Our study contributes to the growing body of literature aimed at deciphering the complexities of SSC biology and the regulation of spermatogenesis in mammalian species, with implications for understanding male fertility.

## Graphical Abstract

**Graphical Abstract:**
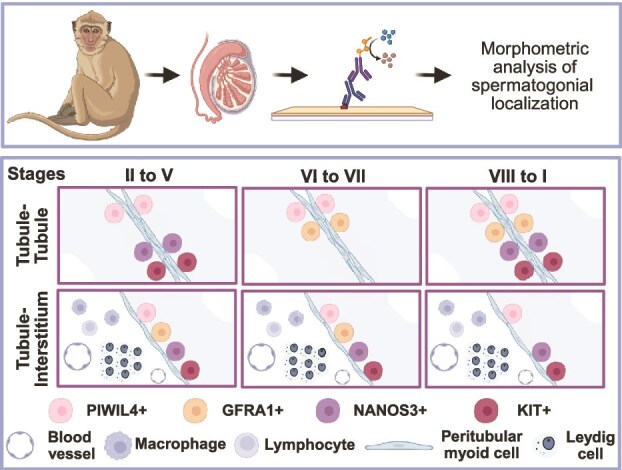

## Teaser

We reveal stage-dependent spatial patterns of spermatogonial subpopulations in *Macaca fascicularis*, uncovering dynamic niche interactions that coordinate spermatogenesis.

## Introduction

As for other self-renewing adult tissues, the function of the seminiferous epithelium relies on the biological activity of resident stem cells, namely the spermatogonial stem cells (SSCs). Like other adult stem cells, SSCs are influenced by various extrinsic signals derived from the “stem cell niche.” The stem cell niche refers to the highly specialized microenvironment, which plays a crucial role in supporting tissue homeostasis by balancing the production of stem cells and progenitor cells. This balance is achieved through cell–cell and cell-matrix interactions, as well as signaling molecules that modulate the transcriptional programs within the cells [1].

The SSC niche is located along the basal lamina of the seminiferous tubules and includes somatic Sertoli cells, which are in close contact with germ cells, and other niche somatic cells outside the seminiferous tubule, such as endothelial cells. Unlike other adult stem cells housed within a “closed” niche, SSCs exist in an “open niche.” According to this model, in mammals, SSCs are intermingled with progenitor and differentiating spermatogonia in the basal compartment of the seminiferous epithelium [2]. Additionally, due to cyclical fluctuations in the expression of paracrine regulators, the nature of the SSC niche changes over the course of the seminiferous epithelial cycle [3]. Thus, SSCs are influenced by both their spatial distribution within the seminiferous tubules and the complex interplay of metabolic and molecular signals.

There is limited knowledge about the exact location of SSCs and the spermatogonial stem niche, and whether these aspects are evolutionarily conserved between rodents and primates. Importantly, although SSCs in mice are formally defined by their functional capacity, in primates SSCs identity is inferred from marker expression, largely based on mouse studies. In rodents, it has been shown that undifferentiated spermatogonia (undiff-SPG), identified based on histomorphological criteria of the nucleus, are not randomly distributed within the seminiferous tubules. At specific stages of the seminiferous epithelial cycle, they are preferentially located in regions of the tubule close to the interstitial areas [4]. These interstitial areas are characterized by a rich vascular network, suggesting that the presence of vessels or the accessibility to vessel-derived morphogens influences the behavior of undifferentiated spermatogonia [5, 6]. Observational studies using an Id4-eGfp transgenic mouse demonstrated that, unlike progenitors and differentiating spermatogonia, the functionally defined ID4-eGFP+ SSC population is typically located in tubular regions that lack contact with the interstitial space [7]. More recently, histological detection of oxygen-sensing probes using Id4-eGfp transgenic mice, revealed that the majority of SSCs are found in low-oxygen environments, with EPAS1 (HIF-2α) playing a key role in sustaining their function within these hypoxic conditions [8]. Additionally, the analysis of single-cell RNA sequencing (scRNA-seq) databases from mouse and human testes has shown that SSCs exhibit a gene expression profile characteristic of glycolytic metabolism whereas differentiating spermatogonia displayed a conserved upregulation of genes related to mitochondrial function, biogenesis, and oxidative phosphorylation [9]. Taken together, these findings suggest that while hypoxia is crucial for maintaining SSCs, the shift from a stem cell state to progenitor and differentiating cells coincides with relocation to areas of the testes with higher oxygen levels, where hypoxia signaling is diminished.

While the localization of the spermatogonial stem niche and the distribution of the most undifferentiated spermatogonia in rodents is under debate, even less is known about primates. Evidence regarding the distribution of potential SSCs in primates comes from Caldeira-Brant and colleagues, who, based on morphological recognition of spermatogonia, proposed that in humans, the reserve stem cells, identified as type A_dark_ spermatogonia with a nuclear vacuole, are preferentially located near blood vessels when they are in a non-proliferative state, as determined by the absence of the mitotic MCM7 marker [10]. This finding supports the hypothesis that seminiferous tubule regions in close contact with the interstitium and blood vessels are favorable niches for primitive spermatogonia.

The development of scRNA-seq and marker analysis clearly showed that undiff-SPG are phenotypically heterogeneous with several subpopulations involved. Interestingly, the transcriptional profile of SPG subpopulations in mice, human and macaque revealed a conserved transcriptional profile across species with several markers commonly expressed in the three species, such as PIWIL4, ID4, GFRA1, LIN28, etc [11]. There is a consensus that PIWIL4+ and GFRA1+ spermatogonia are the most primitive spermatogonia and NANOS3 is expressed by early differentiating spermatogonia (early-diff-SPG) [12]. However, subtle differences in the SPG subpopulation dynamics exist. For example, in rodents MIWI2, the orthologous of PIWIL4, is expressed in a subset of NGN3-expressing transit-amplifying undiff-SPG, downstream of GFRA1 [13], while in primates PIWIL4-expressing SPG have been suggested as the origin of spermatogonial differentiation process [11, 14–16]. We have recently shown that, in Macaca fascicularis*,* the number of the primitive spermatogonia expressing PIWIL4 and/or GFRA1 doesn’t fluctuates significantly between the different stages of the epithelial cycle [17]. Among the undiff-SPG, the only fraction of spermatogonia engaged in the cell cycle is the GFRA1+ spermatogonia subset, suggesting that in adult primates, GFRA1 is required to trigger spermatogonial proliferation and to give rise to spermatogonia committed to differentiation [17, 18]. GFRA1 is the co-receptor for GDNF, one of the best characterized niche components regulating SSCs [3, 19]. Interestingly, we found that distribution of spermatogonia subsets does not fluctuate significantly between stages suggesting that the phenotypic heterogeneity in primitive spermatogonia is not directly correlated to the epithelial stages [17].

Expanding upon this knowledge, our study focuses on elucidating the topological distribution of undiff-SPG and differentiating spermatogonia (diff-SPG) in *M. fascicularis*, a species of macaque commonly used in biomedical research. In most primate, the epithelial stages occupy a small area of the tubule basal lamina and the neighboring area are in randomly different epithelial stage. However, in *M. fascicularis*, the 12 stages of the epithelial cycle are separated along the tubules and occupy large areas of the basal lamina allowing to correlate spermatogonial phenotype to the stages of seminiferous epithelial cycle [20]. To date, the topographical distribution of SPG with respect to the cycle of seminiferous epithelium has been described in rodents, and very limited information is available in primates [4, 5, 10]. Leveraging immunofluorescence (IF) and immunohistochemistry (IHC) techniques combined with histomorphometry analysis, we investigate the distribution undiff-SPG and diff-SPG along the basal compartment across the stages of the seminiferous epithelium cycle, employing specific molecular markers for distinct subpopulations of spermatogonia.

## Materials and methods

### Testicular biopsies

The monkey testicular tissue samples (*M. fascicularis*) were obtained from the breading facility of University of Münster, Germany. Material from six mature animals included in the present study were described in Capponi et al. 2023 [17]. Ethical approval for the use of monkey tissues was obtained according to German federal law on the care and use of laboratory animals (license # 39.32.7.1).

### Immunohistochemistry

Immunohistochemistry (IHC) was performed as described previously on 5 μm-thick Bouin fixed, paraffin-embedded testis sections mounted on polylysine-coated slides [21]. After dewaxing and rehydration, antigen retrieval was performed by incubating the slides in citrate buffer pH 7.8 (UCS diagnostic, Morlupo, Italy) for samples incubated with anti-PIWIL4 or, anti-GFRA1 antibodies, or in citrate buffer pH 6.0 for the samples incubated with anti-NANOS3 or anti-KIT antibodies, in a microwave oven at 750 W, three times for 5 min each (Table 1). For each animal, only one paraffin block was available representing a random part of the testis. Typically, for each marker, we repeated the staining two- or three-times using sections that were collected 20 microns apart in each experiment.

**Table 1:** Table showing all the primary antibodies used in this study

Antibody and Dilution	Species	Company	Product code
PIWIL4 (1:100)	Rabbit	LifesSpan	LS-C482396
GFRA1 (1:30)	Goat	R&D	AF714
NANOS3 (1:100)	Rabbit	Proteintech	21,679–1-AP
cKIT (1:30)	Goat	R&D	AF332
PNA (1:200)		Molecular Probes	L-32458

After the antigen retrieval, endogenous peroxidase was quenched, and nonspecific binding was blocked using Super-Block (UltraTek HRP Anti-Polyvalent kit, ScyTek Laboratories). Sections were incubated overnight at 4°C with the appropriately diluted primary antibody (Table 1). After washing, the sections were processed using the avidin-biotin peroxidase complex (ABC) method, according to the manufacturer’s instructions (UltraTek HRP Anti-Polyvalent kit, ScyTek Laboratories). Negative controls were performed by omitting the primary antibody. Peroxidase activity was visualized using 3,3-diaminobenzidine tetra hydrochloride (Roche Diagnostic, Monza, Italy), and nuclei were shortly counterstained with Mayer hematoxylin (PanReac AppliChem, ITW Reagents). Following washing, the sections were incubated for 2 h at room temperature with fluorescein-conjugated peanut agglutinin (PNA) lectin (1:200, Molecular probes L-32458) for acrosomal staining and then mounted using Vectashield mounting medium. Slides were analyzed using a Zeiss Axioskop 2 light Plus light microscope.

### Identification of the stages of the cycle of the seminiferous epithelium.

Stages identification of testis sections was based on acrosomal development, visualized via PNA immunofluorescence staining [22]. Germ cell nuclear morphology was also used as a parameter to distinguish stages for seminiferous epithelium cycle [17, 18, 23]. In *M. fascicularis* the proportion of single-staged tubules is around 80% and multi-staged cross sections were not included in the study [20]. To ensure sufficient statistical robustness of the analysis, data from two consecutive stages were combined and presented as six different groups (i.e., stages II-III, IV-V, VI-VII, VIII-IX, X-XI, XII-I).

### Quantification and topographical position of spermatogonial subsets

**Figure 1:**
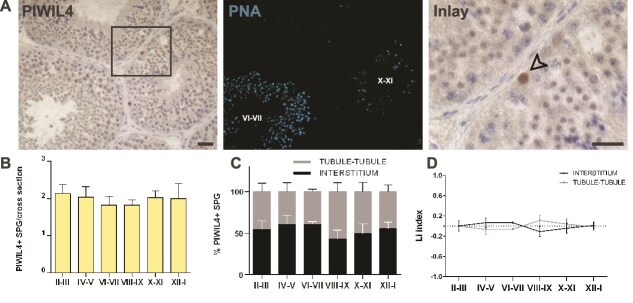
Evaluation of the histological distribution of PIWIL4-expressing spermatogonia (A) Representative images of IHC staining for PIWIL4 (left), and the corresponding IF PNA staining to identify the stage (light blue, middle). The arrow in the inlay indicates PIWIL4+ undifferentiated spermatogonia (right). Scale bars: 100 μm. (B) Quantification of PIWIL4+ undifferentiated spermatogonia per cross-section. A total of 600 cells were scored from n = 3 animals. (C) Percentage of PIWIL4+ undifferentiated spermatogonia opposing the interstitial region or the tubule-tubule region along the seminiferous epithelium cycle. (D) Li graph showing the distribution of PIWIL4+ undifferentiated spermatogonia during the seminiferous epithelium cycle. At least 25 cross-sections were analyzed for each of the 3 animals.

**Figure 2:**
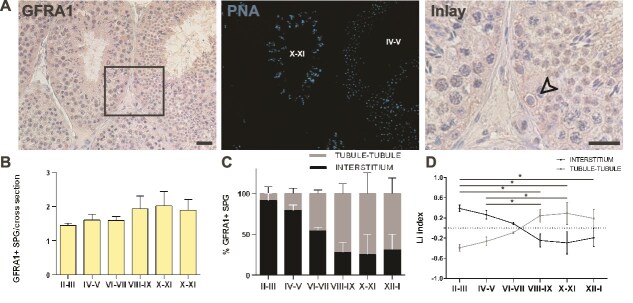
Evaluation of the histological distribution of GFRA1-expressing spermatogonia. (A) Representative images of IHC staining for GFRA1 (left) and the corresponding IF PNA staining to identify the stage (light blue, middle). The arrow in the inlay indicates GFRA1+ undifferentiated spermatogonia (right). Scale bars: 100 μm. (B) Quantification of GFRA1+ undifferentiated spermatogonia per cross-section. A total of 350 cells were scored from n = 3 animals. (C) Percentage of GFRA1+ undifferentiated spermatogonia opposing the interstitial region or the tubule-tubule region along the seminiferous epithelium cycle. (D) Li graph showing the distribution of GFRA1+ undifferentiated spermatogonia during the seminiferous epithelium cycle. At least 20 cross-sections were analyzed for each of the three animals. The statistical comparisons were performed using One-way Anova, followed by post-hoc Tukey test. *P < 0.05.

**Figure 3:**
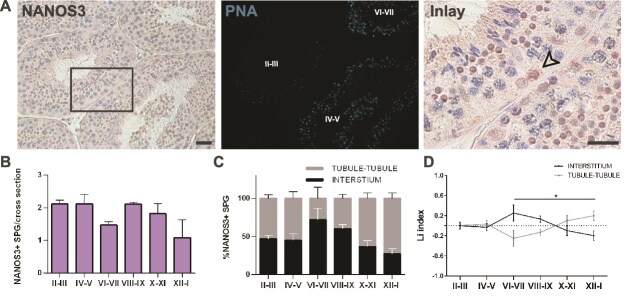
Evaluation of the histological distribution of NANOS3-expressing spermatogonia. (A) Representative images of IHC staining for NANOS3 (left) and the corresponding IF PNA staining to identify the stage (light blue, middle). The arrow in the inlay indicates NANOS3+ early differentiated spermatogonia (right). Scale bars: 100 μm. (B) Quantification of NANOS3+ early differentiated spermatogonia per cross-section. A total of 390 cells were scored from n = 3 animals. (C) Percentage of NANOS3+ early differentiated spermatogonia opposing the interstitial region or the tubule-tubule region along the seminiferous epithelium cycle. (D) Li graph showing the distribution of NANOS3+ early differentiated spermatogonia during the seminiferous epithelium cycle. At least 30 cross-sections were analyzed for each of the three animals. The statistical comparisons were performed using One-way Anova, followed by post-hoc Tukey test. *P < 0.05.

**Figure 4:**
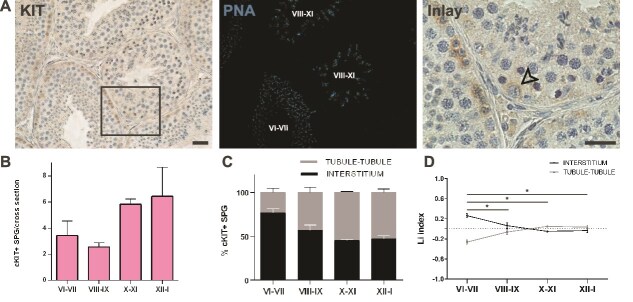
Evaluation of the histological distribution of KIT-expressing spermatogonia. (A) Representative images of IHC staining for KIT (left) and the corresponding IF PNA staining to identify the stage (light blue, middle). The arrow in the inlay shows KIT+ differentiating spermatogonia (right). Scale bars: 100 μm. (B) Quantification of KIT+ spermatogonia per cross-section. A total of 300 cells were scored from n = 3 animals. (C) Percentage of KIT+ differentiating spermatogonia opposing the interstitial region or the tubule-tubule region along the cycle of the seminiferous epithelium. (D) The Li graph for the distribution of KIT+ differentiating spermatogonia during the cycle of the seminiferous epithelium. At least 20 cross sections were analyzed for each of the three animals. The statistical comparisons were performed using one-way ANOVA, followed by post-hoc Tukey test. *P < 0.05.

To quantify the relative proportion of spermatogonial subtypes in the different stages of the cycle, for each marker, testicular sample from three different animals were employed (panel B in Figure 1–4). For the quantification we selected all the single-stage cross sections using the “serpentine” method and for each cross-section, we annotated the number of positive cells and the epithelial stage. The number of positive SPG scored for each marker is indicated in the figure legend. To study the localization of each spermatogonial subpopulation in relation to interstitial and tubule-tubule regions, we employed the method described by Chiarini-Garcia [4, 24]. Briefly, digital micrographs were taken from immunostained slides in regions with well-preserved tissue architecture showing intact tubule-tubule and tubule-interstitial contacts. Quantifications were performed on stored images using ImageJ.

For the Li calculation, two distinct colors were used to trace the perimeter of each tubule cross section on stored images. A grey line was used to draw the portions of the tubule in contact with adjacent tubules (defined as areas where no space was visible between neighboring seminiferous tubules). A black line was used to draw the interface of the tubule and the interstitial space. For each tubular cross section, the total length of each colored line was measured, and the stage of the epithelial cycle was annotated. Next, the number of spermatogonia positive for PIWIL4, GFRA1, NANOS3 or KIT were classified as opposite to the interstitial or tubule contact areas. For each marker, at least 20 cross sections per animal (n = 3 animals) were analyzed.

The Strauss linear selectivity index (Li) was used to assess regional preferences of the various spermatogonial subsets in the basal compartment of seminiferous tubules at each stage [25]. In each staged cross section, the Li was calculated for each tubule-tubule (t–t) and for tubule-interstitial region (t-i):


\begin{align*} Li (t- t) = ri - pi\end{align*}

\begin{align*}Li (t-i) = ri - pi\end{align*}

Where Li is the selectivity index value, ri is the proportion of a given spermatogonial subpopulations in the region, and pi is the proportion of length in the same region. Li values range from −1 (complete avoidance) to +1 (complete preference), with 0 indicating a random distribution. In each animal, two Li values were obtained for each of the six groups of stages (i.e., stages II-III, IV-V, VI-VII, VIII-IX, X-XI, and XII-I): one for the tubule-interstitial region and one for the tubule-tubule region.

### Statistical analysis

All quantitative data are shown as the mean ± SEM. For the Li index analysis, data were analyzed using one-way analysis of variance (ANOVA) followed by a post hoc Tuckey to assess the significance of the differences between many groups (i.e., the stages of the cycle). The significance level was fixed at *P* = 0.05.

## Results

We have recently shown that the number of the primitive spermatogonia expressing PIWIL4 and/or GFRA1 does not fluctuate significantly between the different stages of the epithelial cycle [17]. However, despite their constant number, the topographical localization of early spermatogonia along the basal lamina may differ. We therefore addressed the question whether, during the epithelial cycle, spermatogonia subpopulations are randomly located along the basal lamina or, on the contrary, tend to occupy specific areas. To this end, we performed IHC staining of selected spermatogonia markers followed by fluorescein-conjugated peanut agglutinin (PNA) staining for detection of the stage of the seminiferous epithelium, using *M. fascicularis* testis sections (Supplemental Figure S1).

To assess whether, during the epithelial cycle, spermatogonia subpopulations are randomly distributed, we first analyzed the distribution of PIWIL4+ undiff-SPG (Figure 1). PIWIL4 immunoreactivity was detected in the nucleus of isolated spermatogonia (Figure 1A), and on average, 1.5 PIWIL4+ spermatogonia per cross-section were detected. When considering only tubular cross-section with at least one PIWIL4+ spermatogonia, the average number of spermatogonia positive for PIWIL4 immunoreactivity did not change significantly between the stages of the epithelial cycle (Figure 1B). Evaluation of the percentage of PIWIL4+ spermatogonia localized at tubule-tubule contact areas or facing the interstitial space, across the stages of the seminiferous epithelium cycle revealed an equal distribution between tubule-tubule contact areas and interstitium – facing regions. (Figure 1C). Hence, PIWIL4+ spermatogonia were randomly distributed over the basal compartment for most of the seminiferous epithelium cycle, showing no preference for a given region (Figure 1D).

On average, GFRA1+ spermatogonia were less frequent than PIWIL4+ spermatogonia, with about 1 GFRA1+ spermatogonia per cross-section. Considering solely tubular cross-sections with at least one GFRA1+ spermatogonia, their average number didn’t fluctuate significantly during the seminiferous epithelial cycle (Figure 2B). However, the analysis of the histological distribution revealed that, in contrast to the PIWIL4+ spermatogonia subpopulation, GFRA1+ spermatogonia showed a stage-specific localization (Figure 2C). Li index analysis confirmed that in the first part of the cycle (stage II–VI), GFRA1+ spermatogonia preferentially occupy the areas of tubules facing the interstitial tissue, while they tend to localize at tubule-tubule contact areas in the second half (stages VII–I) (Figure 2D).

To study the histological distribution of spermatogonia committed to differentiation we performed immunohistochemical staining for NANOS3. In positive cells, NANOS3 immunoreactivity was observed in both cytoplasm and nucleus (Figure 3A). Cell quantification revealed an average of 1.3 NANOS3+ spermatogonia per cross-section, with a stage-dependent fluctuation in the number of NANOS3+ spermatogonia per cross-section throughout the seminiferous epithelial cycle (Figure 3B). Analysis of their topographical distribution relative to the basal compartment facing the interstitial or the tubule-tubule contact area revealed a non-random distribution along on the basal lamina at distinct stages of the cycle (Figure 3C), similar to GFRA1+ spermatogonia. At stages VI-VII, NANOS3+ spermatogonia were preferentially localized in interstitium-facing regions, whereas at stages XII-I, they predominantly localized to tubule-tubule contact regions (Figure 3D).

Early-diff-SPG are the progenitor cells that give rise to the first generations of diff-SPG [15]. To assess their spatial distribution, we analyzed the topographical localization of KIT+ spermatogonia from stage VI onward, when type B1 spermatogonia first appear, followed by B2 and B3 [17] (Figure 4A and B). Histological analysis revealed that, the first generation of diff-SPG preferentially localized to the interstitial regions during stages VI-VII, similar as in rodents [4]. In contrast, the B2 and B3 generations were randomly distributed, with no preference for tubule-tubule contact zones or interstitium-facing areas (Figure 4C and D).

## Discussion

In the mammalian testis, undifferentiated and differentiating spermatogonia are found along the basal lamina of the seminiferous tubules. In rodents, it is well established that the localization of spermatogonia along the basal lamina is non-random and varies with the stage of the cycle and the presence of interstitial tissue [4, 5]. In the present study, we provide histological evidence of a non-random distribution of spermatogonial subpopulations during the stages of the seminiferous epithelium cycle in primates. By following the topographical distribution of undiff-SPG along the basal lamina, we found that PIWIL4+ spermatogonia are randomly distributed in all stages of the cycle, i.e., they do not show a preference for regions of the tubule facing other tubules or the interstitial tissue. According to scRNA-seq, PIWIL4 is expressed in the most primitive spermatogonia in primates [12, 15, 16] and altered PIWIL4+ spermatogonia dynamic was linked to male infertility [16, 26]. Since PIWIL4+ spermatogonia are quiescent under normal conditions, their random distribution along the basal lamina, suggests that this subpopulation is not strictly influenced by niche factors. On the contrary, in the first half of the cycle, GFRA1+ spermatogonia are preferentially found in areas of the tubules facing the interstitial tissue while in the second half of the cycle, they preferentially occupy the basal lamina in tubule-tubule contact areas. Considering that we have previously shown that among all undiff-SPG, only those expressing GFRA1 proliferate [17, 18], these data suggest that their relative position along the basal lamina may regulate their proliferation index. It is noteworthy that GFRA1+ spermatogonia predominantly face the interstitial area during stages II-V, when they exhibit a lower proliferative rate, while their proliferative index increases from stages VI to I, as they are located at tubule-tubule interfaces [17]. Interestingly, this observation parallels the findings of Caldeira-Brant and colleagues, who reported that quiescent human A-dark spermatogonia are situated close to blood vessels [10]. This suggests that signals from the interstitial area may keep the GFRA1-expressing spermatogonia adjacent to it in a quiescent state, while those further away are able to proliferate, expanding the pool of SSCs and the spermatogonia primed for differentiation.

NANOS3 is an RNA-binding protein that plays essential roles during male germ cell development [27, 28]. Recently, NANOS3 was identified as a marker for early differentiating spermatogonia by scRNA-seq both in human [15, 16] and monkey [12]. Monocle pseudotime trajectory analysis, which aligns individual cells along a developmental trajectory, showed that early differentiating spermatogonia gives rise to diff-SPG [15]. In the present study, we analyzed the topographical arrangement of both NANOS3+ spermatogonia and the first generation of diff-SPG, namely B1 spermatogonia at stage VI-VII [17]. By IHC analysis we found that NANOS3+ spermatogonia are present at all stages of the seminiferous epithelium cycle. Interestingly, while NANOS3+ spermatogonia are uniformly distributed in most of the stages, at stage VI-VII most of the cells were found close to regions facing the interstitial tissue, similar to B1 spermatogonia, the first generation of diff-SPG. In line with this data in mice, we found that while the first generation of diff-SPG preferentially reside in regions adjacent to interstitial tissue, B2 and B3 spermatogonia are randomly distributed along the basal lamina [4]. These data suggest that the transition between early diff-SPG and diff-SPG occurs in cells facing the interstitial tissue at stage VI-VII.

**Figure 5:**
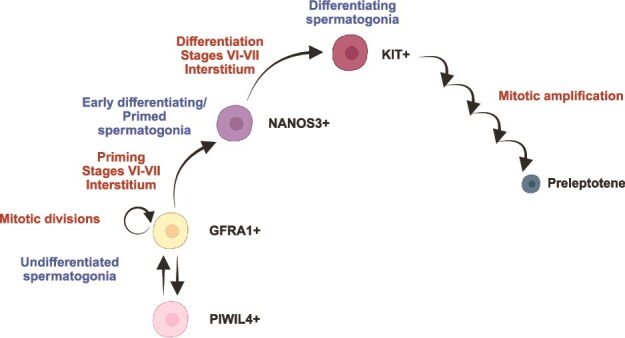
Tentative model for position-dependent spermatogonial progression in adult primate (modified from Capponi et al. 2023). Created with BioRender.com

Analysis of scRNA-seq data from mice and humans showed that diff-SPG upregulate genes associated with mitochondrial function and oxidative phosphorylation, whereas SSCs show a gene expression profile indicative of glycolytic metabolism [9]. This suggests that oxygen levels influence spermatogonial cell fate, associating SSCs with hypoxia and differentiating cells with normoxia. Our data supports the idea that in non-human primates, progenitor and early differentiating spermatogonia are more likely to reside in areas of the basal compartment adjacent to the interstitial space containing the testicular vasculature. Building on these findings, we propose a tentative model for position-dependent spermatogonial progression in adult primates taking into consideration the relationship among spermatogonial subsets during steady-state spermatogenesis and the complex transition highlighted by scRNA-seq studies (Figure 5) [12, 15–17]. We propose that, within the pool of quiescent GFRA1+ spermatogonia located near the interstitial tissue during the first half of the cycle, some are committed to differentiation and eventually become NANOS3+ spermatogonia by stage VI. At stages VI-VII, these early differentiating spermatogonia adjacent to the interstitium eventually acquire KIT expression, leading to the formation of the first generation of differentiating spermatogonia, at stage VII (Figure 5).

The small number of SPG markers (four in this study) and the use of fixed tissues which are unfit to capture dynamic biological process, represent a limitation of the present study. Moreover, descriptive observations can only infer about the topographic localization of SPGs and their developmental potential. However, given that dynamic in vivo lineage tracking or functional assays are not achievable in primates, this study emphasizes the importance of the histological analysis in evaluating whether results from mouse models can be applied to primate and ultimately, to human reproductive biology. Although further studies are necessary to characterize the factors and signals involved, our work adds to the growing body of literature aimed at unravelling the complexities of SSC biology and regulation of spermatogenesis in mammals.

## Supplementary Material

ioag018_Supplementary_Figure_caption

ioag018_Figure_S1

## Data Availability

All raw data are available from the authors upon request.

## Notes

**Grant Support:** This study was supported by Sapienza Università di Roma (RG1221816B8E6ABC) to E.V. and A.F.
