# Supplementary material for: The topographical distribution of spermatogonial subpopulations during the cycle of seminiferous epithelium in Macaca Fascicularis
Source: Biol Reprod. 2026 Jan 21;114(4):1498–506. doi: 10.1093/biolre/ioag018 (PMC13079449; doi:10.1093/biolre/ioag018)
Supplement: ioag018_Supplementary_Figure_caption [file ioag018_supplementary_figure_caption.docx]

**Figure S1.** Acrosome staining by fluorescein-conjugated PNA for detection of the stage of the seminiferous epithelium in testis sections of *Macaca fascicularis*. Upper row: Microphotographs of each of the 12 stages of the cycle (Mayer hematoxylin counterstaining). Lower row: Confocal microscopy images of corresponding PNA staining (red). Inlays show a higher magnification of the spermatid acrosomes detected by PNA staining.
