# Supplementary figures and images for: The topographical distribution of spermatogonial subpopulations during the cycle of seminiferous epithelium in Macaca Fascicularis
Source: Biol Reprod. 2026 Jan 21;114(4):1498–506. doi: 10.1093/biolre/ioag018 (PMC13079449; doi:10.1093/biolre/ioag018)

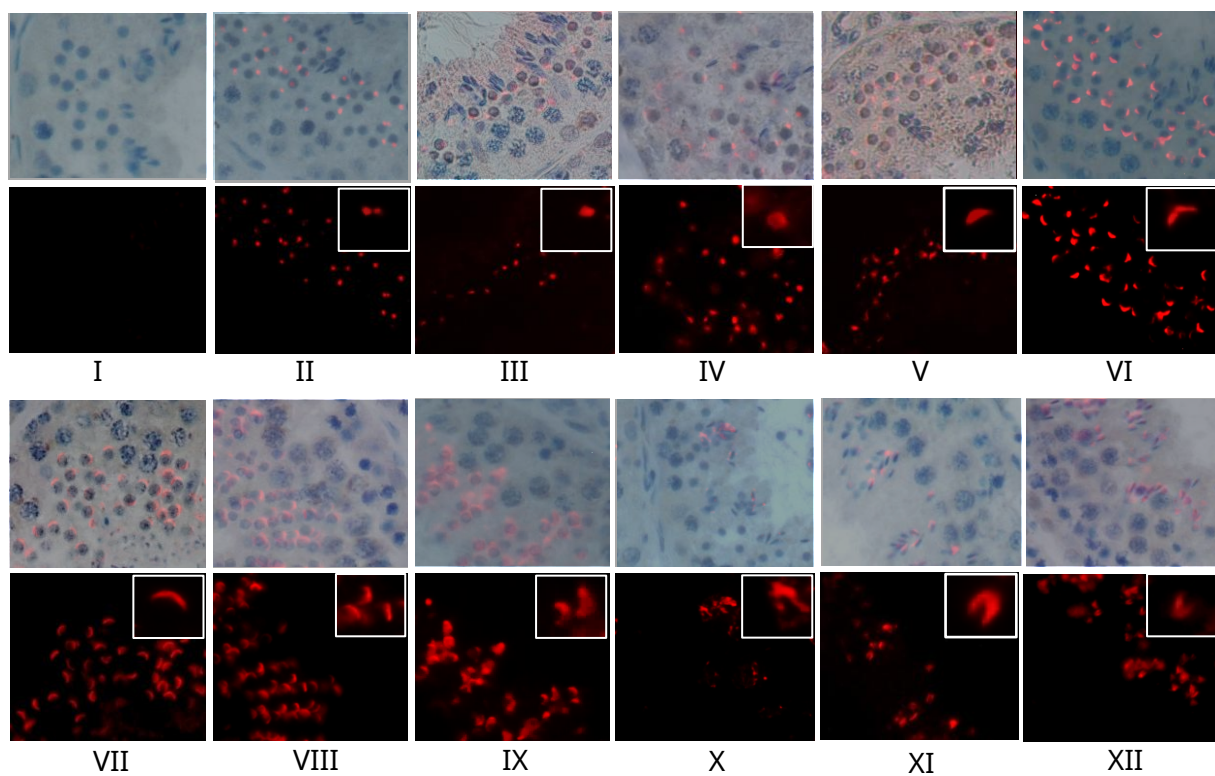

Figure S1

Supplement: ioag018_Figure_S1 [file ioag018_figure_s1.pdf]
